# Joint specific power production in cycling: The effect of cadence and intensity

**DOI:** 10.1371/journal.pone.0212781

**Published:** 2019-02-22

**Authors:** Lorents Ola Aasvold, Gertjan Ettema, Knut Skovereng

**Affiliations:** Centre for Elite Sports Research, Department of Neuromedicine and Movement Science (INB), Faculty of Medicine and Health Sciences, Norwegian University of Science and Technology, Trondheim, Norway; Nanyang Technological University, SINGAPORE

## Abstract

**Background:**

The effect of cadence and work rate on the joint specific power production in cycling has previously been studied, but research has primarily focused on cadences above 60 rpm, without examining the effect of low cadence on joint contribution to power.

**Purpose:**

Our purpose was to investigate joint specific power production in recreational and elite cyclists during low- and moderate cycling at a range of different cadences.

**Methods:**

18 male cyclists (30.9 ± 2.7 years with a work rate in watt at lactate threshold of 282.3 ± 9.3 W) performed cycling bouts at seven different pedalling rates and three intensities. Joint specific power was calculated from kinematic measurements and pedal forces using inverse dynamics at a total of 21 different stages.

**Results:**

A main effect of cadence on the relative to the total joint power for hip-, knee- and ankle joint power was found (all p < 0.05). Increasing cadence led to increasing knee joint power and decreasing hip joint power (all p < 0.05), with the exception at low cadence (<60 rpm), where there was no effect of cadence. The elite cyclists had higher relative hip joint power compared to the recreational group (p < 0.05). The hip joint power at moderate intensity with a freely chosen cadence (FCC) was lower than the hip joint power at low intensity with a low cadence (<60 rpm) (p < 0.05).

**Conclusion:**

This study demonstrates that there is an effect of cadence on the hip- and knee joint contribution in cycling, however, the effect only occurs from 60 rpm and upward. It also demonstrates that there is a difference in joint contribution between elite- and recreational cyclists, and provide evidence for the possibility of achieving higher relative hip joint power at low intensity than moderate intensity by altering the cadence.

## Introduction

The effect of cadence on cycling performance has been studied extensively with a majority of studies focusing on cycling energetics [1]. A number of studies have also focused on the effect of cadence on cycling technique and coordination [2–4]. These studies show that changing cadence leads to numerous technical responses, such as changes in muscle activation and force effectiveness [1, 5, 6].

A technical parameter that could provide insight into the coordination strategy during cycling is the analysis of the relative joint-power distribution. Power delivered to the pedals is mainly produced by muscles that span the ankle-, knee- and hip-joint [7–11]. Several studies have investigated the effect of different factors, among others cadence and work rate, on the joint contribution. There seems to be a consensus that increasing work rate leads to an increase in relative hip joint contribution and a decrease in relative knee joint contribution [8–10].

The consensus is not as clear regarding the effect of cadence. Skovereng et al. [12] and Mornieux et al. [9] both showed that an increase in cadence led to a decrease in relative hip joint contribution and an increase in relative knee joint contribution. In contrast, McDaniel et al. [11] showed no effect of cadence on relative hip and knee joint powers. However, both Skovereng et al. [12] and Mornieux et al. [9] used submaximal intensities and cadences up to 110 rpm while McDaniel et al. [11] used cadences up to 180 rpm at maximal intensity, thus making direct comparisons difficult. The relative ankle joint contribution is reported to be unaffected by cadence [9, 11, 12].

Because cyclists train and compete over a broad range of different cadences and work rates it is important that the interplay between cadence and work rate is understood. Low cadence training is a commonly used mode for elite and recreational cyclists and is generally characterized by moderate intensity and cadences below 60 rpm. Although there are no clear advantages of low cadence training compared to freely chosen cadence when it comes to physiological factors [13–15], the effects of changing cadence invites the investigation of low cadence training from a technical standpoint. Based on the variety of cadences used by cyclists in training and competition, an understanding of the changes in joint specific power over a wider range of cadences than previously studied is needed. This understanding of the effect of cadence on joint specific powers may be useful to researchers, as well as coaches and athletes. For example, if the relationship between hip power and cadence also applies to very low cadences (< 60 rpm), low cadence training at moderate intensity may be regarded as specific training of the hip joint; it would allow the athlete to target specifically the hip muscles, thus mimicking the joint power distribution at high work rate, without needing to work at high intensity. Although low cadence training is done both by recreational and professional cyclists, the majority of studies investigating the effect of cadence on joint specific power are done on recreational athletes [7, 8, 10, 12]. This does not mean that these groups of cyclists, although both trained in cycling, are affected in the same way by extremely low cadences. Therefore, it is also of interest to investigate this effect in two groups of different performance levels.

The aim of the current study was therefore to investigate joint-specific power production in recreational- and elite cyclists during low- and moderate cycling at a range of different cadences. Based on the reviewed literature, we hypothesized that reducing cadences (below 60 rpm) would lead to a further increase in hip joint power and cycling at low cadence and low intensity would lead to a hip joint contribution comparable to moderate intensity and freely chosen cadence (FCC).

## Methods

### Participants

Eighteen well-trained male cyclists, ranging from recreational (n = 9) to elite (n = 9) cyclists (Table 1) were recruited through different cycling clubs in Norway. The recreational cyclists had previous cycling experience, but were excluded if competing at national level. To be included as a elite cyclists, the following criteria was fulfilled: 1) competitive cyclist at UCI-registered Continental team, and 2) competed in 10+ UCI cat.2 races in the previous 12 months. After explaining all procedures, written informed consent was obtained from each subject individually. The study was registered, and approved by Norwegian Social Science Data Services and conducted in accordance with the Declaration of Helsinki.

**Table 1:** Subject characteristics.

	Recreational	Elite
*n*	9	9
Age (years)	39.8 (3.4, 25–51)	22.0 (0.5, 19–24)
Weight (kg)	85.8 (3.3, 73.4–105.3)	73.4 (2.8, 62.4–90.1)
Height (cm)	184.1 (2.0, 174.5–193.0)	182.6 (1.9, 173.0–190.0)
HR_max_ self-reported (bpm)	192.0 (2.0, 180–200)	201.4 (1.6, 190–205)
WR_LT_ (W)	249.9 (6.6, 227.1–286.0)	314.8 (8.0, 278.0–345.2)
WR_LT_ (W/kg)	2.9 (0.1, 2.5–3.5)	4.3 (0.2, 3.7–4.9)
20-min all-out mean (W)	271.9 (10.1, 235–329)	364.1 (8.7, 331–404)
20-min all-out mean (W/kg)	3.5 (0.3, 2.6–4.0)	4.9 (0.2, 4.4–5.6)
Freely chosen cadence (rpm)	86.3 (4.3, 55.3–100.6)	86.5 (3.2, 72.9–95.7)

Mean (SE, range) for subject characteristics. WR_LT_ = work rate in watt at lactate threshold. Mean 20-min all-out power was determined as a self-paced performance test blinded to power utilizing a freely chosen cadence. Freely chosen cadence is defined as the mean cadence at the Int_LT_ with FCC.

### Experimental protocol

The study was conducted as a cross sectional trial, and subjects came to the laboratory for two occasions separated by a maximum of three days. The first day consisted of anthropometric measurements followed by a lactate profile test and a performance test. The protocol started with 20 minutes warm-up with freely chosen cadence and intensity where the athletes were instructed to maintain a low intensity. Following the warm-up a lactate threshold test with 4–7 submaximal, incremental stages starting at 125 W with 50 W increments every 5 minutes until blood lactate exceeded 2 mMol/l were performed. The increments were reduced to 25 W and continued until blood lactate exceeded 4 mMol/l. Blood lactate was measured after 4:30 of each work load, and heart rate (HR) and rate of perceived exertion (RPE) using Borg’s RPE scale [16] at the end of each workload. The work rate corresponding to a blood lactate of 4mmol/L was set as the lactate threshold (LT).

The second day of testing started with 20 min of warm-up at 50% of LT with FCC. This was followed by 30 seconds of cycling at 60 rpm at a workload equivalent to LT. Following 5 minutes of active recovery the main test started with a total of 21 stages at different cadences and intensities each lasting 60 seconds and separated by 30 sec cycling at 50% of LT at a FCC. The cadences used were FCC, 40, 50, 60, 80, 90 and 100 rpm and they were all used at three intensities corresponding to 55% (Int_55_), 85% (Int_85_) and 100% (Int_LT_) of a work rate corresponding to the predetermined LT. The three intensities started with FCC while the remaining 6 cadences were randomized using a cross-over design with one group going from low to high cadence and one group from high to low cadence in order to avoid a potential effect of the order of the included cadences in the main test.

HR and cadence was measured continuously during all stages. Lactate and RPE was measured at the end of the last stage at every work rate. Pedal forces and kinetic variables were measured for 30 seconds of each cadence at all work rates. The subjects were instructed to remain seated with the hands placed on the hoods while performing the intervals. The participants were not informed about when the kinetic data were recorded.

### Equipment and measurements

All measurements were performed in a laboratory with steady conditions (temperature ~22 ^o^C and relative humidity ~45%). All cycling tests were performed on an electronically braked, indoor cycle trainer (CompuTrainer, RacerMate Inc, Seattle, USA) with the participants personal road bicycles. This assures that the cycling technique used by the riders is as authentic as possible and eliminates technical effects resulting from position on the bicycle (i.e., seat height etc.). The cycle trainer was set up and calibrated according to the manufacturer’s instructions. Blood lactate was measured using the Biosen C-Line Sport lactate measurement system (EKF Industrial Electronics, Magdeburg, Germany). Heart rate was measured with a heart rate monitor (Polar M400, Polar Electro OY, Kempele, Finland).

Cycling kinematics was measured using an eight-camera Oqus motion capture system (Qualisys, Sweden) using a sample rate of 100 Hz. Reflective markers were placed on the neck (cervical spine), pelvis (iliac crest), hip (greater trochanter), knee (lateral epicondyle), ankle (lateral malleolus) and on the front and back of custom made extensions placed symmetrically on the pedal axis. The kinematic data was collected from both limbs and pedals, and average values were used.

Pedal forces were measured with two custom pedals equipped with two force cells (Revere Model 9363, capacity 250 kg per cell, the Netherland) at a sample rate of 100 Hz. A description of the force pedal system and calibration procedures can be found in Ettema et al. [17].

### Data analysis

Cycling kinematics where collected using Qualisys Track Manager software (Qualisys, Sweden) which allowed for the integration of the analog pedal force signals and thus, simultaneous recording of the two. Using Matlab R2015b, joint powers for the hip, knee and ankle joints were calculated using inverse dynamics for a linked system of rigid segments [17–19]. In short, the powers at the joints are calculated from the pedal forces, the movements of the body segments and the inertial estimates (mass and moment of inertia) of these segments, by applying Newton’s inertial laws. Guidelines for calculating masses and moments of inertia were taken from Van Soest et al. [20].

### Statistical analysis

All descriptive data are presented as mean ± standard error. Where applicable, 95% confidence intervals (CI) are reported. A linear mixed model was used to evaluate the effect of cadence, intensity and athlete level on joint specific power. Because external power was not always identical at the different cadences, relative (normalized for total joint power) rather than absolute joint power was used as a covariant in the analyses. Fishers LSD analysis was used to localise and evaluate the content of the effect of cadence and intensity and effect size was calculated as partial Eta squared for the main joint power analysis. When an effect of athlete level was found a one-way ANOVA was used to evaluate the effect. Statistical significance was accepted at p < 0.05. All data analysis and statistical analysis was conducted using SPSS 24.0 (SPSS, Chicago, USA) for mac and Matlab R2015b and R2016b (MathWorks Inc. Natic, USA).

## Results

**Fig 1:**
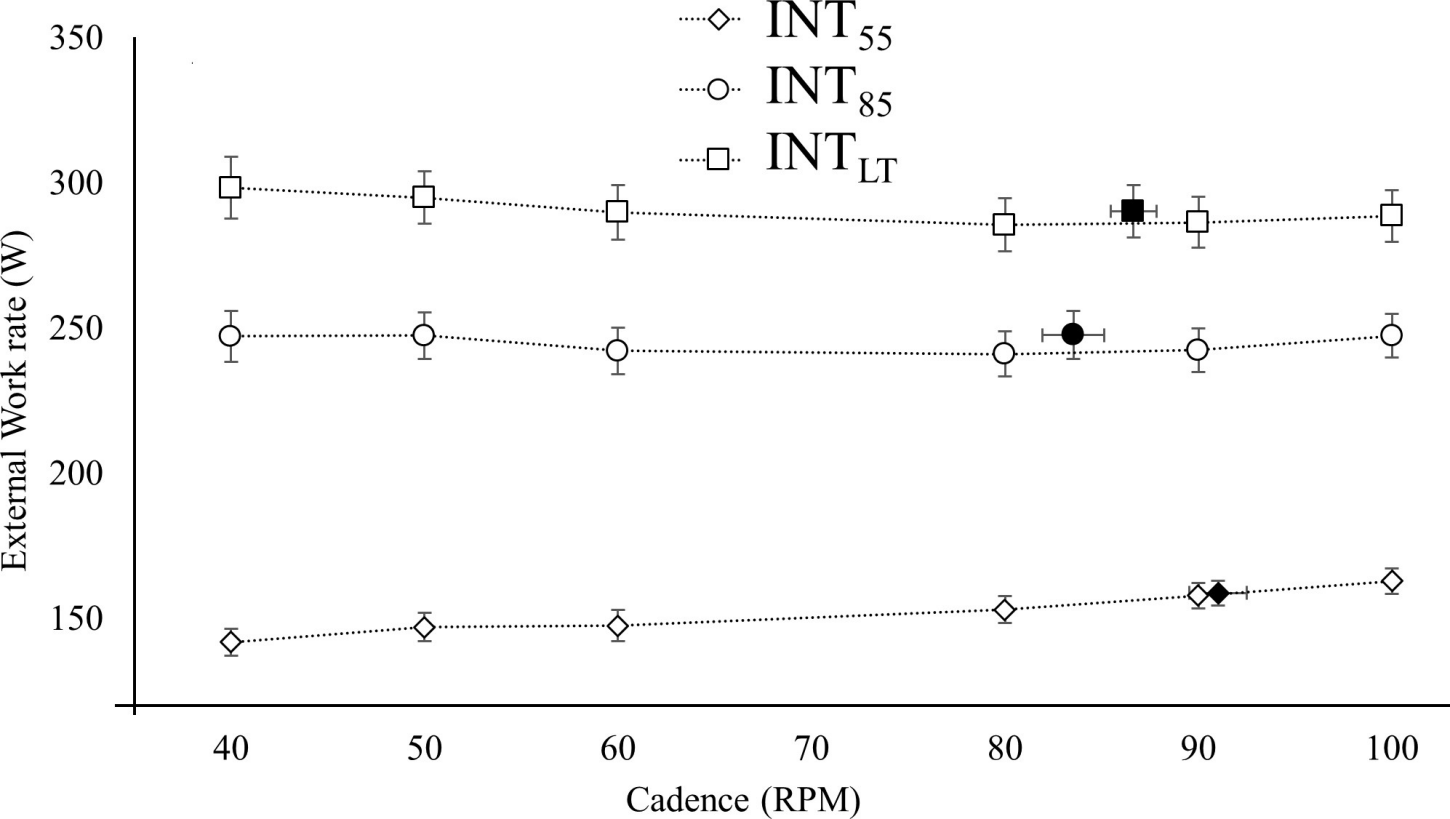
External power against cadence. Group mean and standard error for (A) external power at Int_55_ (diamond), Int_85_ (circle) and Int_LT_ (square) at all cadences (filled marker indicate FCC).

The mean external work rate calculated from the pedal forces for all cadences were 152.6 ± 8.8 W at Int_55_, 245.0 ± 8.2 W at Int_85_ and 290.4 ± 9.6 W at Int_LT_. Fig 1 shows the external power against cadence. The different cadence conditions led to differences in the external work rate of 6.2 ± 0.8, 2.1 ± 0.1 and 3.4 ± 0.6 W at Int_55_, Int_85_ and Int_LT_ respectively.

### Effect of cadence

**Fig 2:**
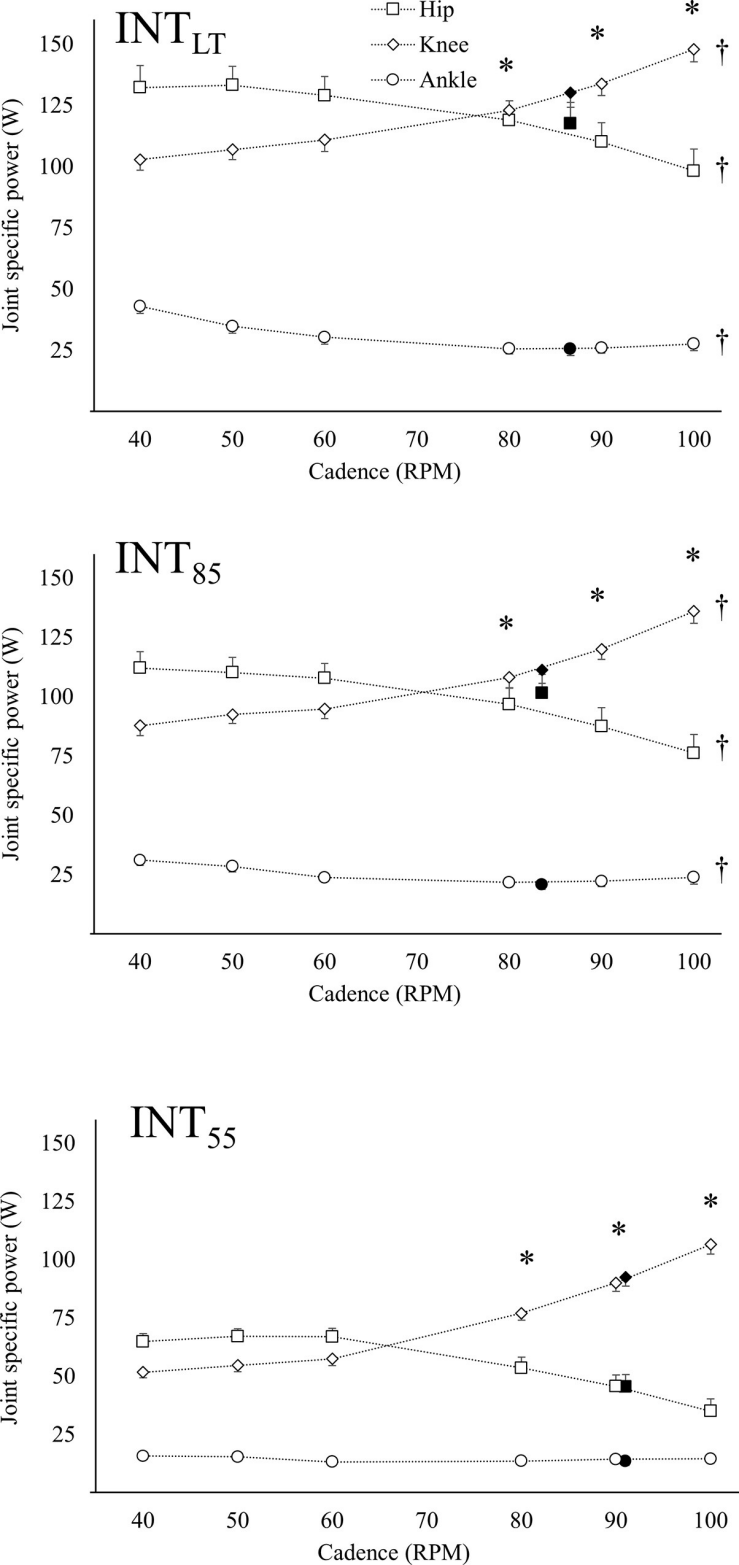
Total joint power for hip, knee and ankle joint actions. Group mean and standard error for total joint power in hip (square), knee (diamond) and ankle (circle) joint at Int_55_, Int_85_ and Int_LT_. Filled marker indicate FCC. * indicate a difference in joint power from previous cadence for the hip and knee joints. Daggers indicate a difference in the mean total joint power from previous intensity.

**Fig 3:**
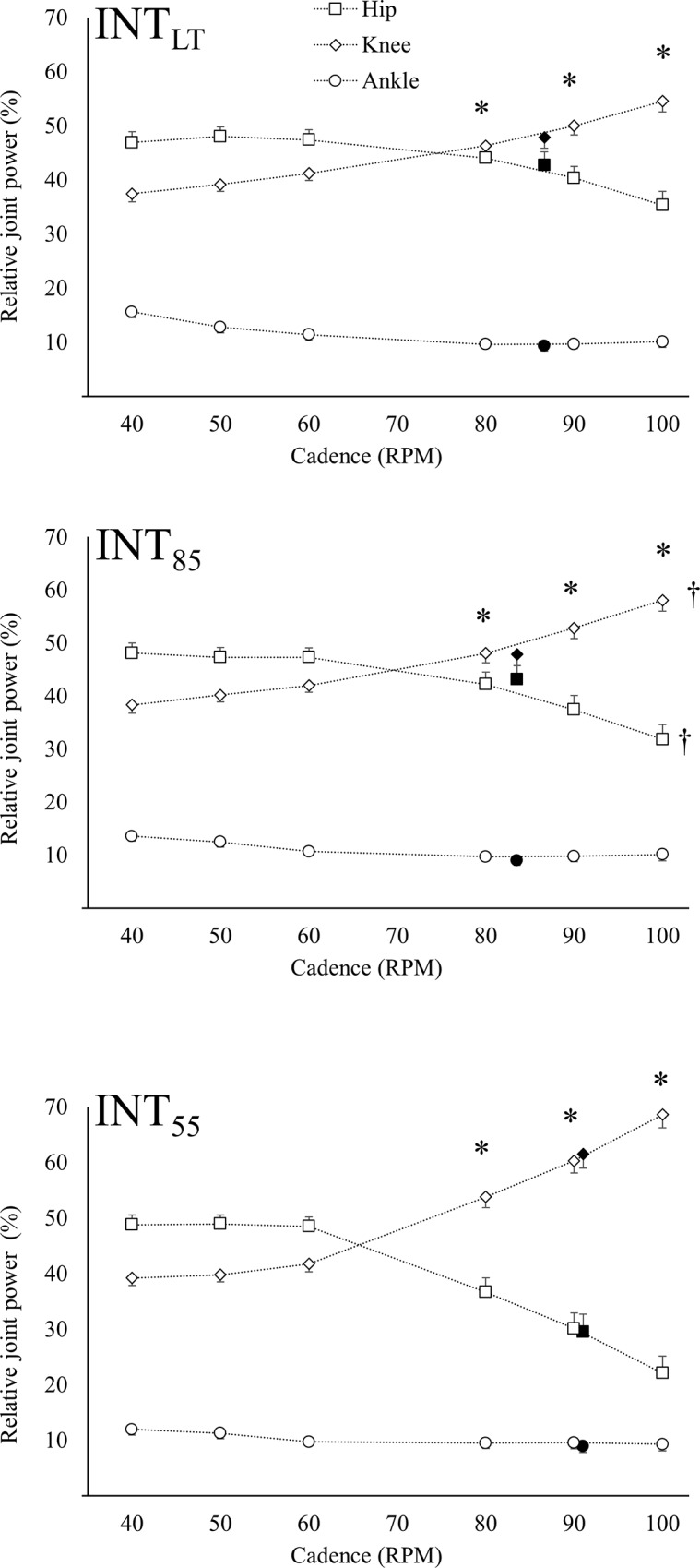
Relative joint power for hip, knee and ankle joint actions. Group mean and standard error for relative joint power in hip (square), knee (diamond) and ankle (circle) joint at Int_55_, Int_85_ and Int_LT_. Filled marker indicate FCC. * indicate a difference in joint power from previous cadence for the hip and knee joints. Daggers indicate a difference in the mean relative joint power from previous intensity.

Absolute- and relative joint power at different cadences is presented in Figs 2 and 3, respectively. The joint power ranged from 34.9 to 134.9 W for the hip joint, 51.6 to 147.9 W for the knee joint and 13.2 to 42.9 W for the ankle joint. For Int_55_, the hip joint was the main power producing joint at low cadence (≤60 rpm) (p < 0.05), while the knee joint was the main power producing joint at high cadence (≥80 rpm) (p < 0.05) but there was no difference at 60 rpm (p < 0.09). A similar joint contribution was seen at Int_85_ and Int_LT_ where the hip joint being the main power producing joint at low cadence (≤50 rpm) (p < 0.05) and the knee joint was the main power producing joint at high cadence (≥90 rpm) (p < 0.05).

A main effect of cadence on the relative hip- [F(5,306) = 33.86, p < 0.001, partial eta-sq = 0.89], knee- [F(5,306) = 76.00, p < 0.001, partial eta-sq = 0.93] and ankle joint power [F(5,306) = 8.12, p < 0.001, partial eta-sq = 0.84] was found. In general, an increase in cadence led to a decrease in relative hip joint power and an increase in relative knee joint power. However, from 60 rpm and below there was no significant effect of cadence. For the relative ankle joint power, a trend of decreased ankle joint contribution with increased cadence was found, with a significantly lower ankle joint contribution at high cadence (≥80 rpm) compared to low cadence (≤50 rpm).

### Effect of intensity

A main effect of intensity on the relative hip- [F(2,306) = 6.16, p < 0.001, partial eta-sq = 0.71] and knee joint contribution [F(2,306) = 16.87, p < 0.001, partial eta-sq = 0.90] was found. The relative hip joint power at low intensity (Int_55_) was lower than at moderate intensity (Int_85_ and Int_LT_), while there was no significant difference between Int_85_ and Int_LT_. An increase in intensity led to a decrease in relative knee joint power between low (Int_55_) and moderate (Int_85_ and Int_LT_) intensity, with no effect between Int_85_ and Int_LT_. There was no effect of intensity on the relative ankle joint power.

An interaction effect of intensity and cadence was found for both hip- [F(10,306) = 2.30, p < 0.05, partial eta-sq = 0.91] and knee joint contribution [F(10,306) = 2.91, p < 0.01, partial eta-sq = 0.95]. Contrast analysis revealed a smaller effect of changing cadence at Int_LT_ compared to Int_55_ (Fig 3). No interaction effect was found for the ankle joint contribution.

### Sub-group analysis

In order to investigate the effect of performance level, we divided the cohort into elite and recreational level cyclists. The elite cyclists had higher WR_LT_ and 20-min all-out power compared to the recreational cyclists, with 315 ± 7.6 and 364 ± 7.8 W compared to 250 ± 6.5 and 272 ± 9.2 W respectively. There was no difference in the freely chosen cadence between the groups (p > 0.19).

Overall, the whole group results were also seen in both subgroups with an increase in cadence leading to an increase in knee joint contribution and decrease in hip joint contribution (p < 0.01) and an increase in work rate led to increased hip joint contribution and decreased knee joint contribution (p < 0.01). There was no difference in the effect of work rate or cadence between the groups (both p > 0.53) but there was a difference in the relative joint distribution where the elite cyclists overall had a 9.8 percentage higher relative hip joint contribution (p < 0.01).

## Discussion

The purpose of this study was to investigate joint-specific power production during low- and moderate cycling at a range of different cadences and additionally to investigate differences in the effect of cadence and work rate between elite and recreational cyclists. The main findings of this study were that an increase in cadence leads to a decrease in relative hip joint power and an increase in relative knee joint power. However, the effect of cadence on the relative hip- and knee joint power only occurred from and above 60 rpm.

We performed the statistical analysis on the two identifiable subgroups (recreational versus elite). Although the statistical power for these analyses was limited, the same conclusions could be drawn on the main findings. Our group of participants had a relatively large variation in performance level. If we use the classification of cyclists by Jeukendrup et al. [21] the recreational group are categorized as trained- or well-trained cyclists, while the elite group are categorized as elite based on training and race status. This strengthens the notion that our findings are highly generalizable. However, a main difference between groups was that the elite cyclist showed a higher relative hip power and lower relative knee power compared to the recreational cyclist across all intensities and cadences.

### Effect of cadence

Increasing cadence led to a decreased relative hip joint power and increased relative knee joint power and our findings complies with earlier research [9, 12]. However, contrary to our hypothesis, there was no effect at cadences below 60 rpm. Skovereng et al. [12] and Mornieux et al. [9] both found increasing hip joint power and decreasing knee joint power with decreasing cadence but neither of the aforementioned studies investigated cadences below 60 rpm. The current results indicate that there is a point where a further decrease in cadence produces no change in the joint power distribution.

We hypothesized that cycling at low cadence and low intensity would lead to a hip joint contribution comparable to moderate intensity with a FCC. Our results showed that the hip joint power at low cadence (≤60 rpm) and low intensity was higher than the hip joint power at moderate intensity with a FCC. These results show the possibility of achieving higher hip joint contribution at a lower intensity by altering cadence. This enables a potential for specific hip training without needing high whole-body intensity.

Our results show that there is a shift from the hip joint being the most power producing joint at low cadence (≤60 rpm) to the knee joint being the most power producing joint at high cadence (≥80 rpm).

The elite group had higher relative hip joint contribution and lower relative knee joint contribution compared to the recreational group. Numerous studies have provided evidence that repeated performance of a movement task could facilitate neuromuscular adaptations, which could result in a more skilled movement [22, 23]. Chapman et al.’s [24] findings suggest that highly trained cyclists exhibit more skilled muscle recruitment as a result of neuromuscular adaptations compared to novice cyclists. The differences between highly trained- and novice cyclists found by Chapman et al. [24] likely reflect continued adaptation with long-term training. The difference in joint power between the recreational- and elite cyclists in our study could possibly be an outcome of task experience and movement skill among the athletes. Interestingly, this difference does not seem to have any impact on the cadence effect on the use of hip and knee in power production.

### Effect of intensity

The results regarding the effect of intensity on relative hip-, knee- and ankle joint power complies with previous research [8, 9]. However, there was no significant effect of increasing intensity from Int_85_ to Int_LT_ on the relative hip- and knee joint contribution. Similar results were also found by Elmer et al. [10] who found no effect of increasing external work rate from submaximal to maximal on the relative hip extension power in cyclists. This may indicate that there is an upper limit for the effect of intensity on relative joint contribution, similar as the one found for cadence [11]. To date there are no studies that include a wide enough range of different work rates to conclude on the matter. Taken together, the current findings indicate that an increase from low- to moderate intensity leads to a shift in technique with a greater contribution from the hip joint and decreasing contribution from the knee joint. However, the effect of increasing intensity from moderate to high and maximal is unclear.

There was a variation in the measured work rate at the different cadences caused by difficulties with the resistance on the stationary trainer particularly at Int_55_. The largest discrepancy from the mean work rate occurred at 40 rpm and 100 rpm (10.8 W and 10.2 W respectively) at Int_55_. For the higher work rates, there were minor differences in measured work rate (i.e. average deviation from mean work rate 2.5 ± 0.3 W and 3.4 ± 2.5 W at Int_85_ and Int_LT_ respectively) between the different cadences and should therefore have a minimal impact on the results.

## Conclusion

The present study demonstrates that increasing cadence leads to a decrease in relative hip joint power and an increase in relative knee joint power, however, the effect of cadence only occurs from and above 60 rpm. The study also provides evidence for the possibility of achieving higher hip joint power at low intensity as at moderate intensity by altering the cadence. The findings from the present study provide further knowledge about the effect of cadence and intensity on the joint power contribution among cyclist. These results may have implications for researchers, coaches and athletes in the field of cycling.

## Supporting information

S1 File. Data Dataset for the present publication. (CSV)
